# Oxygen-Resistant Electrochemiluminescence System with Polyhedral Oligomeric Silsesquioxane

**DOI:** 10.3390/polym11071170

**Published:** 2019-07-10

**Authors:** Ryota Nakamura, Hayato Narikiyo, Masayuki Gon, Kazuo Tanaka, Yoshiki Chujo

**Affiliations:** Department of Polymer Chemistry, Graduate School of Engineering, Kyoto University, Katsura, Nishikyo-ku, Kyoto 615-8510, Japan

**Keywords:** electrochemiluminescence, POSS, ruthenium(II) complex, oxygen resistance

## Abstract

We report the oxygen-resistant electrochemiluminescence (ECL) system from the polyhedral oligomeric silsesquioxane (POSS)-modified tris(2,2′-bipyridyl)ruthenium(II) complex (Ru-POSS). In electrochemical measurements, including cyclic voltammetry (CV), it is shown that electric current and ECL intensity increase in the mixture system containing Ru-POSS and tripropylamine (TPrA) on the indium tin oxide (ITO) working electrode. The lower onset potential (*E*_onset_) in CV is observed with Ru-POSS compared to tris(2,2′-bipyridyl)ruthenium(II) complex (Ru(bpy)_3_^2+^). From the series of mechanistic studies, it was shown that adsorption of Ru-POSS onto the ITO electrode enhances TPrA oxidation and subsequently the efficiency of ECL with lower voltage. Moreover, oxygen quenching of ECL was suppressed, and it is proposed that the enhancement to the production of the TPrA radical could contribute to improving oxygen resistance. Finally, the ECL-based detection for water pollutant is demonstrated without the degassing treatment. The commodity system with Ru(bpy)_3_^2+^ is not applicable in the absence of degassing with the sample solutions due to critical signal suppression, meanwhile the present system based on Ru-POSS was feasible for estimating the amount of the target even under aerobic conditions by fitting the ECL intensity to the standard curve. One of critical disadvantages of ECL can be solved by the hybrid formation with POSS.

## 1. Introduction

Electrochemiluminescence (ECL), also called as electrogenerated chemiluminescence, is light emission from the excited species generated through multiple redox reactions on an electrode [1,2]. ECL has many advantages as a platform of sensing materials. Owing to unnecessary light excitation, background noise in the luminescence detection can be drastically reduced by removing scattering of incident light. Compared to chemiluminescence which is produced through chemical reactions, it is relatively easy to achieve temporal and spatial control for emission generation. Therefore, the ECL techniques are recognized as a powerful analytical tool and are used in various areas, such as immunoassay, genosensors, and for monitoring small molecules [1,2,3,4,5,6]. Although a variety of substances can potentially work as a source of ECL, ruthenium(II) (Ru(II)) complexes, especially tris(2,2′-bipyridyl)ruthenium(II) (Ru(bpy)_3_^2+^), are conventionally used because of many superior properties, such as high chemical stability, recyclability, redox properties, and long lifetime in the excited state [2,3,4]. Meanwhile, oxygen is well known to be a crucial quencher to the photo-excited state of Ru(II) complexes [7,8,9], followed by the reduction of the ECL intensity [10,11,12]. Thereby, pretreatments for deoxygenation with the analytical samples are essential before conventional ECL detection. Hence, improvement of oxygen resistance of the ECL system is strongly needed not only for improving accuracy and sensitivity in detection but also for extending applicability to future sensing technologies such as real-time monitoring with the native samples.

Polyhedral oligomeric silsesquioxane (POSS) attracted attention as an “element-block” which is the minimum unit containing heteroatoms for constructing functional materials [13,14]. We report unique and useful properties of POSS-containing hydrophilic materials [15,16,17,18,19]. In aqueous media, it was shown that the water-soluble POSS-containing materials can efficiently capture various types of guest molecules in the distinct hydrophobic spaces created around the POSS units and are applicable for sensors [20,21,22,23,24]. It is proposed that the cubic core is favorable for adsorbing various guest molecules with hydrophobic interaction. In particular, it is shown that the captured molecules in the POSS-core dendrimer are protected from photobleaching. It should be noted that stimuli responsiveness is compatibly observed from the captured guest molecules, although these molecules are isolated by POSS. Because of the steric structure of POSS, accessibility of reactive oxygen species which are promised to induce degradation can be lowered. Meanwhile, energy and electron transferring is acceptable through the POSS scaffold. Owing to these characters, environment-responsive photon upconversion including multiple energy transferring steps [23] and photo-triggered redox reactions [24] are accomplished in the POSS-containing materials.

Inspired by the enhancement of environmental resistance of POSS without losses of stimuli responsiveness, we presume that oxygen resistance in ECL systems might be reinforced. On the basis of this assumption, we synthesize the modified POSS tethered to the tris(2,2′-bipyridyl)ruthenium(II) complex (Ru-POSS) and evaluate the influence of dissolved oxygen on the ECL properties in the aqueous mixture system containing tripropylamine (TPrA, coreactant). Initially it is shown that ECL intensity and electric current can be enlarged by the connection to POSS. Moreover, from the comparison studies, it is clearly indicated that ECL quenching by oxygen is suppressed in the Ru-POSS/TPrA system. We elucidate how this phenomenon could originate from the improvement of TPrA oxidation by Ru-POSS adsorption onto the ITO electrode. Finally, we also accomplish the ECL-based detection for the water pollutant without the degassing pretreatment. The oxygen-resistance enhancement and target detection which tend to show the trade-off relationship are compatible by employing the POSS element-block.

## 2. Results and Discussion

### 2.1. Characteristics of Ru-POSS

**Scheme 1:**
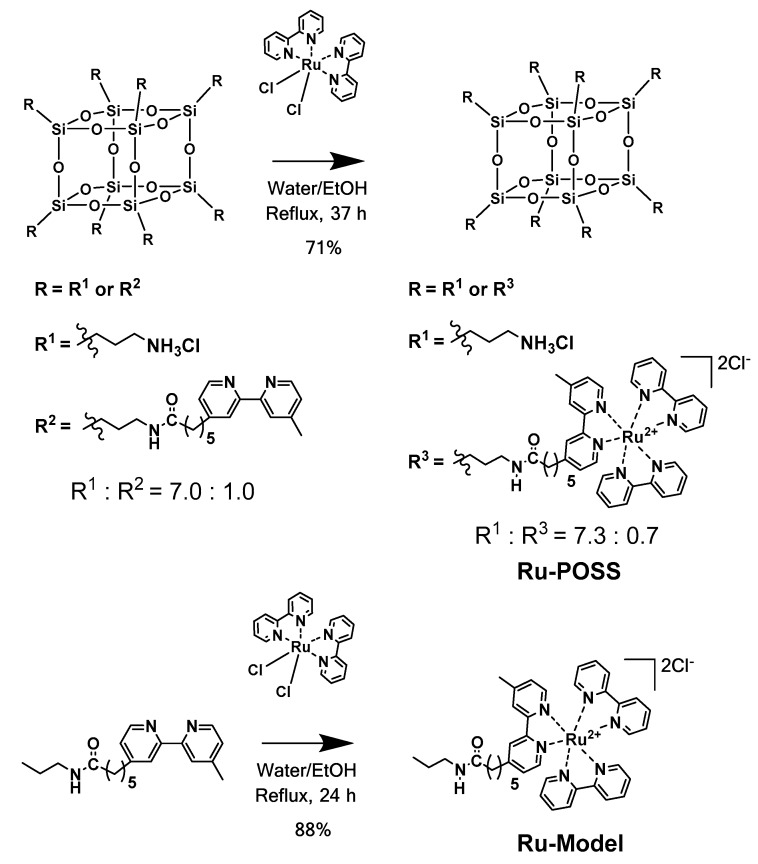
Syntheses of the Ru-POSS and the Ru-Model.

The chemical structures and preparation of the Ru(II)-containing materials involving Ru-POSS and the model compound, Ru-Model are shown in Scheme 1. As is often the case with luminescent dyes, concentration quenching is occasionally induced through non-specific intermolecular interaction in the accumulation of the chromophores. To avoid reduction of sensitivity, we designed Ru-POSS to have the single Ru(II) complex. To obtain the target material, we used octa-substituted ammonium POSS (Amino-POSS) as a scaffold and connected it to the bipyridine ligand for complexation with the Ru(II) ion [24]. After complexation with the commodity reaction condition, the introduction ratio of the Ru(II) complex to the POSS unit in Ru-POSS was estimated from the integration ratios of the signal peaks from the side chains in the ^1^H NMR spectrum. Purification with Ru-POSS was only precipitation and Ru-POSS was obtained as mixtures containing variable numbers of the Ru complex. The structures of synthesized compounds were confirmed by ^1^H, ^13^C and ^29^Si NMR spectroscopies and high-resolution mass spectrometry (HRMS). The detailed procedures are shown in the Supporting Information. The structures of the compounds used, such as Ru(bpy)_3_^2+^, Ru(bpy)_2_(dmbpy)^2+^ (dmbpy = dimethylbipyridine) and tripropyl amine (TPrA), are listed in Figure S1.

### 2.2. ECL Properties of Ru(II)-Containing Materials

**Figure 1:**
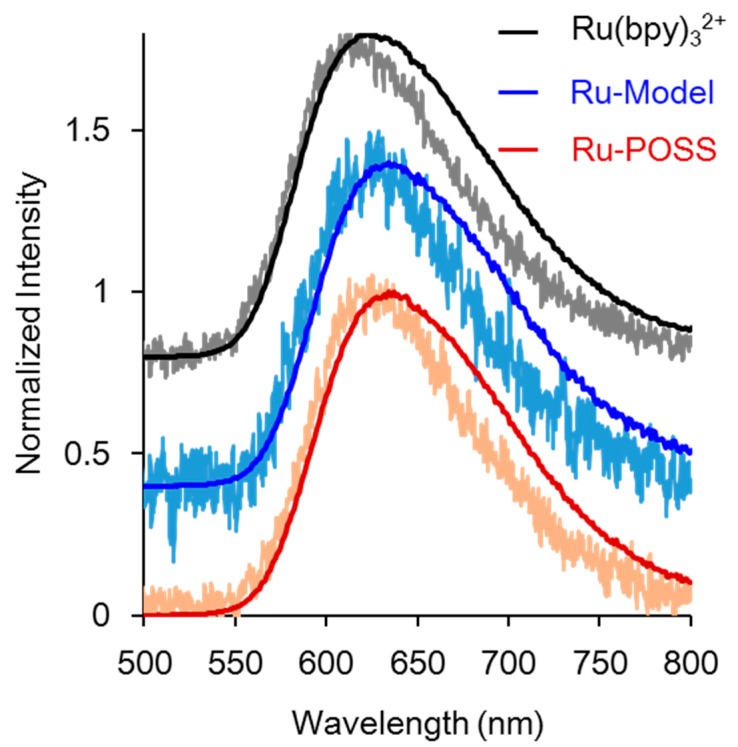
ECL (jagged line) and PL spectra (smooth line) of the Ru(II)-containing materials. The spectra of Ru(bpy)_3_^2+^ and Ru-Model are shifted in the *y*-axis direction for clarity.

Optical measurements were performed to evaluate the influence on the electronic structure of the Ru complex moiety by the connection with POSS (Figure S2). ECL and photoluminescence (PL) spectra of the materials are shown in Figure 1 and Table 1. All compounds containing Ru(II) exhibited the emission bands at similar positions, indicating that ECL originated from the metal-to-ligand charge transfer transition in the triplet excited state (^3^MLCT) in the Ru(II) complex moiety [2,3,4]. By subtracting TPrA or Ru(bpy)_3_^2+^, critical reduction of electric currents was obtained (Figure S3), supporting the conclusion that ECL is produced from the redox cycles including the Ru(II) complex and TPrA through the several proposed mechanisms (Scheme S1). The increase in the Ru(bpy)_3_^2+^ concentration was minimally influenced on the enhancement of ECL intensity (Figure S4). Concentration quenching could occur under condensed conditions. Slight bathochromic shifts were observed in UV−vis absorption and ECL spectra of the solutions containing Ru-POSS and Ru-Model compared to the ECL spectrum of Ru(bpy)_3_^2+^. It is likely that a substituent effect is responsible for these negligible changes [25]. From these results it was shown that electronic structure at the Ru(II) complex moiety is hardly influenced by connecting to the POSS unit.

**Figure 2:**
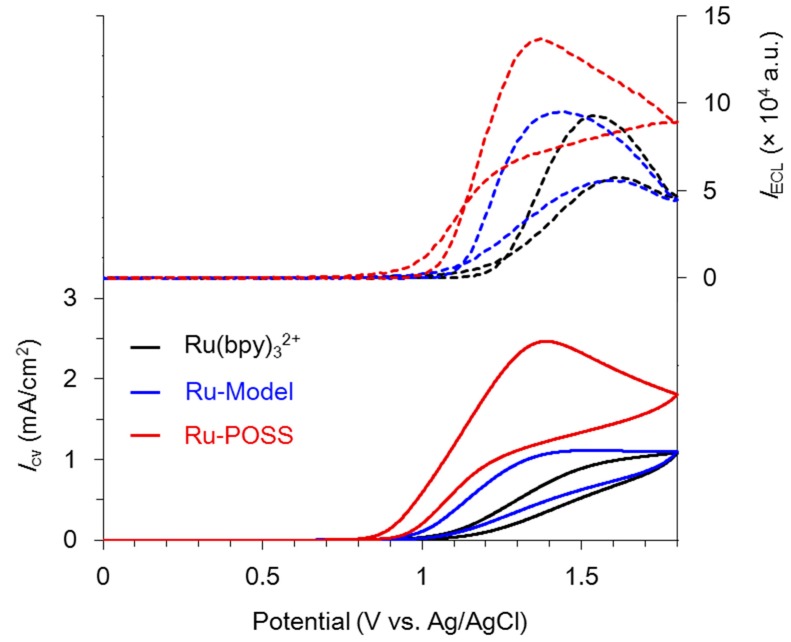
Cyclic voltammograms (solid lines) and corresponding ECL profiles (dashed lines) of 0.1 mM Ru(II) complexes and 100 mM TPrA in 0.20 M PBS buffer (pH 8.8) with an ITO electrode at a scan rate of 100 mV/s (*n* = 3). The concentration of Ru-POSS was based on the Ru(II) complex unit.

The cyclic voltammograms and corresponding ECL profiles in the TPrA co-reactant system are shown in Figure 2. The POSS unit in Ru-POSS induced significant changes in electric and optical properties. There are two advantageous issues resulting from the connection of the Ru(II) complex to POSS. First, it was found via Ru-POSS that ECL appeared in the lower voltage region. HOMO energy levels were estimated from the onset values (*E*_onset_) in the oxidative wave of the CV curves (Figure S5). Obviously, the *E*_onset_ of Ru-POSS decreased compared to the Ru-Model, as well as Ru(bpy)_3_^2+^. Although it was reported that the substituent effect can decrease *E*_onset_ [26], the degree of change was several times larger by the connection to POSS than those in the previous work. In our ECL system, another pathway for decreasing *E*_onset_ could exist. Additionally, even in the absence of TPrA, lower *E*_onset_ values were obtained from the Ru(II)-containing materials except for Ru(bpy)_3_^2+^ (Figure S6), proposing that the lowering value of *E*_onset_ might be induced by changes in molecular morphology on the electrode. The discussion will be made in a later section. Second, a larger anodic current and ECL intensity were obtained from Ru-POSS than those from Ru(bpy)_3_^2+^ and Ru-Model. To understand the roles of POSS in these changes, further experiments were executed.

In the bulk electrolysis (BE) at 1.2 V vs. Ag/AgCl, it was also observed that a larger current was able to be generated from Ru-POSS than those of Ru(bpy)_3_^2+^ and Ru-Model (Figure S7). In particular, the initial non-faradaic current was one order magnitude larger. To explain these behaviors, including lower *E*_onset_, we initially investigated the possibility that Ru-POSS could adsorb at the electrode surfaces and accelerate the redox reactions. To evaluate the validity of this speculation, we examined BE with Ru(bpy)_3_^2+^ in the presence of Amino-POSS under the same condition as above (Figure S8). It was clearly indicated that the connection to POSS was essential for obtaining a larger ECL. Next, the electrodes modified by immobilization of Ru(II) complexes were prepared, and their properties were examined. The ITO electrode was immersed in the ECL reaction solution containing the series of Ru(II) complexes, such as Ru-POSS, Ru(bpy)_3_^2+^ and Ru-Model, rinsed with deionized water and dried in vacuo. Then, the BE and ECL properties were evaluated in the buffer solution containing only TPrA without adding Ru(II)-containing molecular species in the solution (Figures S9–S11). The reduction in current intensity and disappearance of ECL were observed in the ITO electrodes treated with Ru(bpy)_3_^2+^ and Ru-Model, while a large current and ECL were still detected in the Ru-POSS-immersed ITO electrode. This data clearly indicated that the adsorption of Ru-POSS occurred on the ITO electrode. It has been reported that ammonium groups can form tight interaction to the ITO electrode from the studies regarding self-assembling monolayers [27,28,29], poly-l-lysine [30,31,32], and cytochrome C [33]. It is likely that the multiple ammonium groups in Ru-POSS play a critical role in adsorption to the electrode. Moreover, from the adsorption experiments under variable pH conditions, enhancement of ECL was observed by using the modified ITO electrode immersed with Ru-POSS solution in an acidic condition (pH 5.2), supporting the conclusion that the ammonium-ITO interaction is responsible for anchoring Ru-POSS onto the electrode surface (Figure S12). The assembly of functional groups into the compact space as well as the high symmetry of POSS might be favorable for tight interaction.

Regarding the lowering *E*_onset_, it was reported that modification of ITO electrodes with CNT [34], poly(amidoamine) (PAMAM) dendrimer [35], Au nanoparticles [36], and thin film consisting of vertically aligned silica mesochannels [37] lowered the onset potentials and enhanced ECL intensities by facilitating the redox cycles at the electrode surfaces. Similar to these modifications, it is proposed that Ru-POSS could promote the redox cycles for the generation of ECL. Bard et al. reported about the influence of molecular morphology on intensity. They claimed that some degree of distance would be needed for obtaining emission from the Ru(II) complex adsorbed to electrodes, otherwise direct quench by the electrode would occur [38]. From our experiments, although POSS could induce adsorption of Ru(II) species on the electrode, larger ECL was obtained. It is implied that steric hindrance of the silica cube might prevent quenching by the electrode.

### 2.3. Resistance to Oxygen Quenching

**Figure 3:**
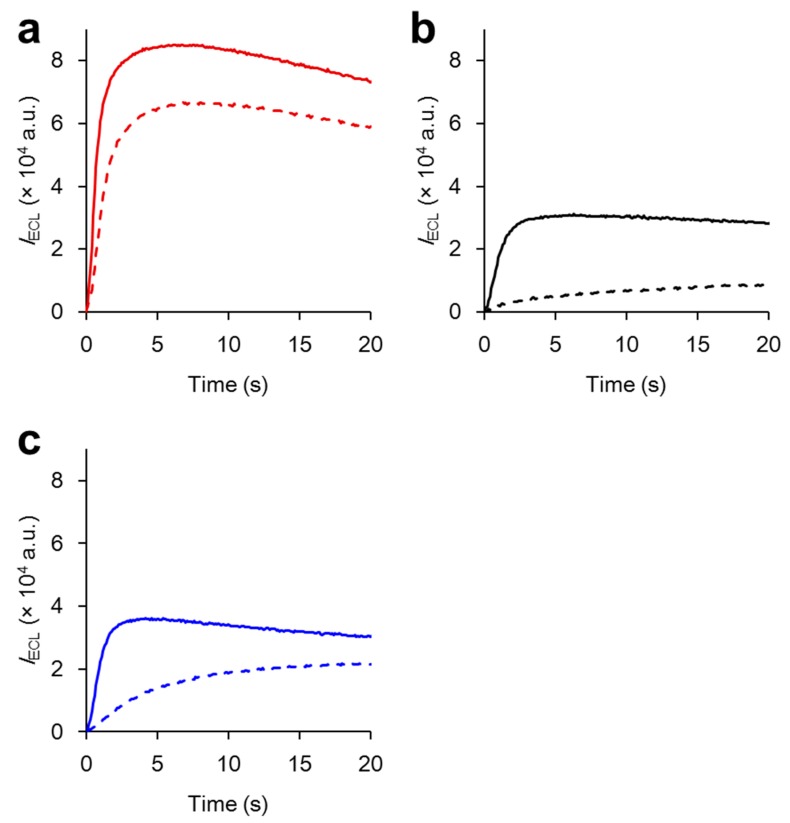
Time courses of ECL with (**a**) Ru-POSS, (**b**) Ru(bpy)_3_^2+^ and (**c**) Ru-Model in BE with 0.1 mM Ru(II) complexes and 100 mM TPrA in 0.20 M PBS buffer (pH 8.8) with Ar (solid line) and O_2_ bubbling (dashed line) with an ITO electrode at 1.2 V vs. Ag/AgCl. The concentration of Ru-POSS was based on a Ru(II) complex unit.

Oxygen resistance of this ECL system was evaluated (Figure 3 and Table 2). To examine the influence of oxygen on the luminescent and electric properties, the dissolved oxygen (DO) levels in the samples were tuned by argon (DO: less than 1 mg/mL) and oxygen bubbling (ca. 20 mg/mL) before monitoring. As readily expected, the aerated sample containing Ru-complex showed critical annihilation of ECL prepared by oxygen bubbling, whereas significant influence were hardly observed in the electric currents (Figure S13). It is well known that DO readily facilitates non-radiation decay of the triplet-excited state, resulting in emission annihilation. In contrast, it was shown that the intensity level from the hypoxic sample containing Ru-POSS was maintained at the higher DO level. These data clearly indicated that the connection of Ru-complex to POSS was responsible for improving oxygen resistance in the ECL system.

To obtain further insight regarding the detailed mechanism and the role of POSS in enhanced oxygen resistance, we performed measurements under various conditions. It was shown that the connection of the Ru(II) complex was necessary for expressing oxygen resistance in the former section (Figure S8 and Table S1). From the PL measurement with the Ru-POSS sample under the aerated condition, it was found that emission annihilation was induced (Figure S14), meaning that oxygen access for the Ru(II) complex moiety could be maintained even in the presence of POSS. Therefore, it was proposed that the rate of TPrA oxidation could be significantly enhanced in the Ru-POSS system. The pH effect on ECL of Ru-POSS was initially investigated (Figure S15 and Table S2). The ECL intensities of all Ru(II)-containing materials drastically decreased under acidic conditions. It should be emphasized that even in the Ru-POSS system, which had high resistance against DO, a lower intensity was shown at pH 5.2 than that at pH 8.8. According to previous literature, deprotonation in TPrA followed by oxidation of TPrA [39,40,41] could be suppressed under acidic conditions. Therefore, the redox cycle for generating ECL should be inhibited. It is already known that there are several pathways in the ECL mechanism of Ru(bpy)_3_^2+^/TPrA system (Scheme S1) [12,40,41,42,43,44]. In these mechanisms, the TPrA oxidation should be the critical step before presenting luminescence. Furthermore, it was reported that the excess amount of TPrA radicals (TPrA^•^) generated by the TPrA oxidation can contribute to reducing of the amount of DO near the ECL reaction layer and subsequently can suppress ECL quenching [12]. In summary, the enhancement of the TPrA oxidation by Ru-POSS might be responsible for improving oxygen resistance in the ECL system as well as ECL intensity.

### 2.4. Analysis of Antibiotics without Deoxygenation

**Figure 4:**
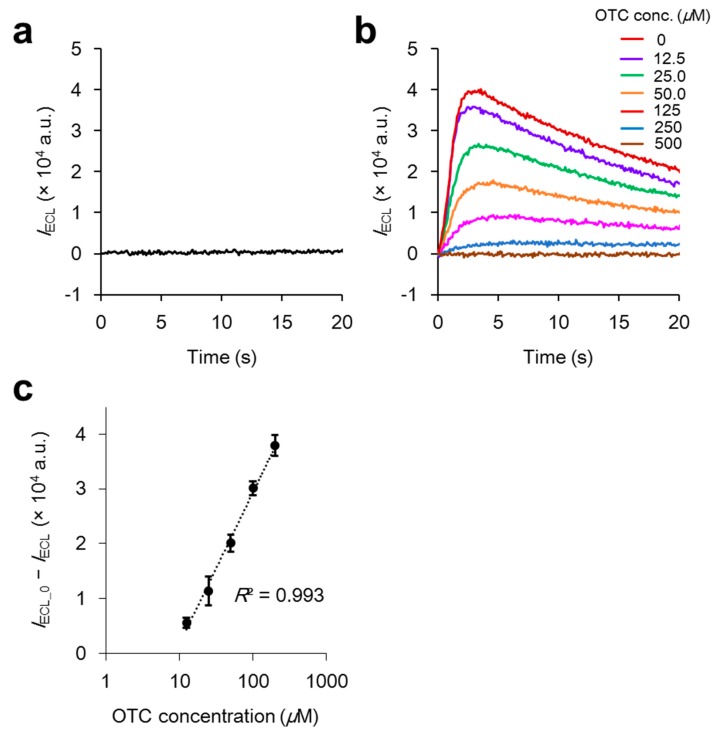
Time courses of ECL with (**a**) Ru(bpy)_3_^2+^ and (**b**) Ru-POSS in BE of 0.1 mM Ru(II) complexes and 100 mM TPrA and various concentrations of OTC in 0.20 M PBS buffer (pH 8.8) stirred for 20 min with an ITO electrode at 1.2 V vs. Ag/AgCl. (**c**) Standard curve prepared from the data from (**b**) (*I*_ECL_ and *I*_ECL_0_ represent the maximum ECL intensity in the time course of BE with and without OTC, respectively).

Because ECL is usually obtained from the triplet-excited state, most conventional ECL systems always suffer from emission quenching by DO in the pristine sample. Therefore, degassing for lowering DO levels in the sample was previously needed in practical usage. To demonstrate the advantage of oxygen-resistant ECL based on Ru-POSS, we finally performed the detection without degassing as a pre-treatment. By evaluating the decrease in ECL intensity, the target concentration was determined [45,46,47,48,49]. By adding oxytetracycline (OTC), which is an anti-bacterial agent for domestic animals that often induces water pollution near livestock barns, to the samples, the changes in ECL intensity were monitored. The OTC aqueous solution was added to the buffer containing 0.1 mM Ru(II) complex and 0.1 M TPrA with stirring and then ECL measurements in BE (1.2 V vs. Ag/AgCl) of the mixtures were taken (Figure 4). Apparently, ECL intensity from the Ru(bpy)_3_^2+^/TPrA system was too small to discriminate the target, meanwhile critical decrease in emission intensity was detected from the Ru-POSS sample, meaning that the existence of OTC was able to be detected even in the aerobic sample. In particular, the linear relationship between *ΔI*_ECL_ and the concentration in the range from 1.3 × 10^−5^ to 2.0 × 10^−4^ M (*R*^2^ = 0.993) was obtained when using Ru-POSS. The limit of detection was 9.8 × 10^−6^ M (S/N = 3). It was suggested that ECL quenching may be caused by oxidized OTC at the electrode, which is a benzoquinone derivative [45]. Benzoquinone groups are well known to have the ability to quench ECL [48,49]. The decay of the excited state of the Ru(II) complex and TPrA intermediate-radical quenching were induced by benzoquinone and the latter pathway was predominant when TPrA direct oxidation occurred [49]. Regarding the detection limit, it has been reported that the OTC concentrations in wastewater are in the range between 10^−6^ and 10^−9^ M order [47]. The detection limit of our system was not so high compared with the other ECL system [45], however, our method could be directly applicable for detecting serious contaminated conditions.

## 3. Conclusions

We demonstrate the Ru(II) complex-modified POSS and its application for oxygen-resistant ECL system. By employing POSS, various beneficial effects were obtained. Electronic currents and ECL intensities were enhanced in the electro- and optical measurements, and *E*_onset_ in CV was lowered compared to Ru(bpy)_3_^2+^. Moreover, ECL quenching by oxygen was suppressed in the Ru-POSS/TPrA system. From our mechanistic studies, adsorption of Ru-POSS to the ITO electrode, followed by efficient TPrA oxidation on the ITO electrode were suggested. Finally, oxygen quenching was compensated by generation of the TPrA radical. Our findings and materials could be a scaffold for developing future sensing technologies based on ECL, such as real-time monitoring for small molecular pollutants which it is difficult to detect with conventional environment sensors. Furthermore, compared to the conventional system, the oxygen-resistance can be improved by connecting to POSS at this stage. As mentioned in the introduction, POSS has significant properties for capturing hydrophobic molecules under aerobic conditions. In combination with the unique capturing property of the POSS unit, improvement of sensitivity and selectivity for the target detection might be proposed.

## Figures and Tables

**Table 1:** Luminescent properties of the Ru(II)-containing materials *^a^*.

Compounds	*λ*_abs_LC_ (nm)	*λ*_abs_MLCT_ (nm)	*λ*_em_PL_ (nm) *^b^*	*Φ*_PL_ (%) *^c^*	*τ* (*µ*s) *^d^*	*λ*_em_ECL_ (nm)	Intensity (×10^4^ a.u.) *^e^*
Ru-POSS	286	456	634	5.6	0.44	607	13.5
Ru-Model	286	456	632	5.8	0.43	631	9.5
Ru(bpy)_3_^2+^	286	454	623	6.3	0.50	632	8.8

*^a^* Based on the concentration of the Ru(II) complex unit (1.0 × 10^−5^ M). *^b^* Excited at *λ*_abs_MLCT._ *^c^* Calculated as an absolute value. *^d^* Measured with the excitation light at 375 nm. *^e^* Determined at the peak value in CV experiments.

**Table 2:** Residual rates of maximum ECL intensities and onset increase rates in BE in the aerated solutions compared to the hypoxic ones.

Compounds	*I*_ECL_^Max^__O2_/*I*_ECL_^Ma^x__Ar_ (%)	*m*_ECL_^Max^__O2_/*m*_ECL_^Ma^x__Ar_ (%)
Ru-POSS	78	51
Ru(bpy)_3_^2+^	38	9
Ru-Model	61	13

*m*: Slope of onset intensity curve.

## Supplementary Materials

The following are available online at https://www.mdpi.com/2073-4360/11/7/1170/s1, Figure S1: Structures of Ru(bpy)_3_^2+^, Ru(bpy)_2_(dmbpy)^2+^ and TPrA, Figure S2: (a) UV−vis absorption and (b) PL spectra of 1.0 × 10^−5^ M solutions containing Ru(bpy)_3_^2+^, Ru-Model and Ru-POSS in H_2_O. The excitation light at *λ*_abs___MLCT_ was used for PL measurements. Inset figure in (a) is the inset around the MLCT band. The concentration of Ru-POSS was based on the Ru(II) complex unit, Figure S3: Cyclic voltammograms of 0.10 mM Ru(bpy)_3_^2+^ and/or 0.1 M TPrA in 0.20 M PBS buffer (pH 8.8) at ITO electrode at a scan rate of 100 mV/s (n = 3), Figure S4: Time courses of ECL in BE of 0.50 mM Ru(bpy)_3_^2+^ and 100 mM TPrA in 0.20 M PBS buffer (pH 8.8) with deoxygenation (solid line) or aeration (dashed line) with an ITO electrode at 1.2 V vs. Ag/AgCl, Figure S5: The determination of onset potential (*E*_onset_). The onset potentials were determined from the intersection of the tangents between the baseline and the signal current, Figure S6: Cyclic voltammograms of 0.10 mM Ru(II)-containing materials in 0.20 M PBS buffer (pH 8.8) with an ITO electrode at a scan rate of 100 mV/s (n = 3). The concentration of Ru-POSS was based on the Ru(II) complex unit, Figure S7: Initial time courses of current in BE of 0.10 mM Ru(II) complexes and 100 mM TPrA in 0.20 M PBS buffer (pH 8.8) at ITO electrode at 1.2 V vs. Ag/AgCl (n = 3). The concentration of Ru-POSS was based on the Ru(II) complex unit, Figure S8: Time courses of ECL with 0.1 mM Ru(bpy)_3_^2+^, 0.1 mM Amino-POSS and 100 mM TPrA in 0.20 M PBS buffer (pH 8.8) with Ar (solid line) and O_2_ bubbling (dashed line) with an ITO electrode at 1.2 V vs. Ag/AgCl, Figure S9: Time courses of ECL in BE of 100 mM TPrA in 0.20 M PBS buffer (pH 8.8) with the modified ITO electrodes at 1.2 V vs. Ag/AgCl, Figure S10: Cyclic voltammograms (solid lines) and corresponding ECL curves (dashed lines) at a scan rate of 100 mV/s with 0.1 mM Ru-POSS and 100 mM TPrA in 0.20 M PBS buffer (pH 8.8) with an ITO electrode (Red line) and 100 mM TPrA in 0.20 M PBS buffer (pH 8.8) with the modified ITO electrode (Blue line). The concentration of Ru-POSS was based on the Ru(II) complex unit, Figure S11: Time courses of currents with (a) Ru-POSS (b) Ru(bpy)_3_^2+^ (c) Ru-Model in BE of 100 mM TPrA in 0.20 M PBS buffer (pH 8.8) with (solid line) or without 0.10 mM Ru(II) complexes (dashed line) at ITO electrode at 1.2 V vs. Ag/AgCl (n = 3). The concentration of Ru-POSS was based on the Ru(II) complex unit, Figure S12: Time courses of ECL in BE of 100 mM TPrA in 0.20 M PBS buffer (pH 8.8) at 1.2 V vs. Ag/AgCl with the modified ITO electrodes immersed into the solution containing 0.1 mM Ru-POSS (based on the Ru(II) complex unit) with 100 mM TPrA in 0.20 M PBS buffer with variable pH values (pH 5.2: orange line and pH 8.8: blue line), Figure S13: Time courses of electric currents with (a) Ru-POSS, (b) Ru(bpy)_3_^2+^, (c) Ru-Model and (d) Ru(bpy)_3_^2+^ + Amino-POSS in BE of 0.10 mM Ru(II) complexes and 100 mM TPrA in 0.20 M PBS buffer (pH 8.8) with Ar (solid line) and O_2_ bubbling (dashed line) with and ITO electrode at 1.2 V vs. Ag/AgCl. The concentration of Ru-POSS was based on a Ru(II) complex unit. The concentration of Amino-POSS in (d) was adjusted to the same as the POSS unit in (a), Figure S14: Residual rates of emission intensities of the photo-excited Ru(II) complexes in aerated solutions consist of 0.01 mM Ru(II) complexes and 0.1 M TPrA in 0.2 M PBS solution (pH 8.8). Excitation wavelengths were *λ*_abs_MLCT_ Residual rates were calculated from the equation, *I*_PL_Air_/*I*_PL_Ar_, where *I*_PL_Air_ is the intensity at *λ*_em_^max^ in aerated solutions and *I*_PL_Ar_ is in the hypoxic solutions, Figure S15: Cyclic voltammograms (solid lines) and corresponding ECL curves (dotted lines) of 0.1 mM Ru(II) complexes and 100 mM TPrA in 0.20 M PBS buffer (pH 5.2) with an ITO electrode at a scan rate of 100 mV/s (n = 3). The concentration of Ru-POSS was based on a Ru(II) complex unit, Table S1: Residual rates of maximum ECL intensities and onset increase rates in BE in the aerated solution compared to the hypoxic one, Table S2: *E*_onset_ in various conditions. Scheme S1: The summary of the ECL mechanisms in the Ru(bpy)_3_^2+^/TPrA system (TPrA^●+^ = Pr_3_N^●+^, TPrA^●^ = Pr_2_NC^●^HCH_2_CH_3_, P_1_ = Pr_2_N^+^CHCH_2_CH_3_).
