# Supplementary material for: Oxygen-Resistant Electrochemiluminescence System with Polyhedral Oligomeric Silsesquioxane
Source: Polymers (Basel). 2019 Jul 10;11(7):1170. doi: 10.3390/polym11071170 (PMC6680606; doi:10.3390/polym11071170)
Supplement: Supplementary file 1 [file polymers-11-01170-s001.pdf]

Supporting Information

Oxygen-resistant Electrochemiluminescence System with Polyhedral Oligomeric Silsesquioxane

Ryota Nakamura, Hayato Narikiyo, Masayuki Gon, Kazuo Tanaka* and Yoshiki Chujo

Department of Polymer Chemistry, Graduate School of Engineering, Kyoto University, Nishikyo-ku, Katsura, Kyoto 615-8510, Japan.

E-mail: tanaka@poly.synchem.kyoto-u.ac.jp

General. Octa-substituted ammonium POSS (Amino-POSS), dichlorobis(2,2'-bipyridine)ruthenium(II) ($Ru(bpy)_2Cl_2$) were prepared according to the previous reports.[1] 5-Bromovaleronitrile, LDA (1.5 M solution in THF/ethylbenzene/heptane) and oxytetracycline (OTC) hydrochloride were obtained from Tokyo Chemical Industry co., Ltd. DMSO-d_6 and CD_3OD were obtained from Euriso-Top. Deionized water was purified with an Autopure WEX3 system from Yamato Scientific Co., Ltd. All other reagents were obtained from Wako Pure Chemical Industries, Ltd.

Chemical structure characterization measurements. ^{1}H NMR, ^{13}C NMR and ^{29}Si NMR spectra were measured with a JEOL EX-400 (400 MHz for ^{1}H) spectrometer or a JEOL JNM-AL400 (400 MHz for ^{1}H, 100 MHz for ^{13}C, 80 MHz for ^{29}Si) spectrometer. Coupling constants (J value) are reported in Hertz. The chemical shifts are expressed in ppm downfield from tetramethylsilane, using residual dimethyl sulfoxide (δ = 2.50 in ^{1}H NMR, δ = 39.5 in ^{13}C NMR) or methanol (δ = 3.31 in ^{1}H NMR, δ = 49.0 in ^{13}C NMR) as an internal standard. ESI MASS spectra were obtained on a Thermo Fisher Scientific MS: Exactive HPLC: Accela 600series. CryoSpray MASS spectra were obtained on a Bruker microTOF II.

Optical properties measurements. UV–vis absorption spectra were recorded with a SHIMADZU UV–vis–NIR Spectrometer UV-3600 at 25 °C. Emission spectra from the samples were monitored using a HORIBA Fluorolog-3 at 25 °C using 1 cm path length cell.

Electrochemical and ECL measurements. Cyclic voltammetry (CV) and bulk electrolysis (BE) were performed with an ALS Electrochemical Analyzer Model 600D (BAS Inc., Japan). A conventional three-electrode method was adopted with a Pt wire as the counter electrode and an Ag/AgCl (saturated with 3 M NaCl) as the reference electrode. The working electrode was indium tin oxide (ITO) electrode (<10 Ω/square, 7 mm × 50 mm). ITO electrodes were cleansed by sequential sonication for 30 min each in the following solvents; ion-exchanged water (IEW) with detergent, acetone, IPA, IEW and then dried *in vacuo*. The sample solutions other than oxygen resistant tests were purged with Ar for 20 min before measurements. In oxygen resistant experiments, the samples were aerated by bubbling for 20 min. Electrochemical cell was kept in the dark, and the ECL concomitant with electrochemical analyses were measured with a

Modular USB Spectrometers USB 4000 (Ocean optics, Inc., USA). ECL intensity was calculated by integration from 550 nm to 800 nm wavelength.

Synthetic procedures and characterization

Preparation of bipyridine ligand containing POSS (bpyL-POSS). BpyL-POSS was prepared according to the previous report.[1] The typical procedure is shown here. 4,4′-dimethyl-2,2′-bipyridine (5.2 g, 28 mmol) was dissolved in THF (100 mL) at room temperature and then cooled to −78 °C. 19 mL of LDA (1.5 M solution in THF/ethylbenzene/heptane) was added to the solution and stirred for 1 h at −78 °C. 5-Bromovaleronitrile (3.6 mL, 31 mmol) in THF (5 mL) was subsequently added, and the reaction mixture was stirred for 3 h at 0 °C. The resulting mixture was diluted with water (100 mL), neutralized with 1 M HCl and extracted with ethyl acetate. The organic phase was washed with brine, dried over $MgSO_4$ and concentrated in *vacuo*. The crude product was dissolved with chloroform and purified by silica gel column chromatography (gradient elution, hexane/ethyl acetate, from 10/1 to 1/1 v/v containing 1 v/v % triethylamine) to give 2.8 g (38%) of 4-(5-cyanopentyl)-4′-methyl-2,2′-bipyridine as a brown powder. A mixture of 4-(5-cyanopentyl)-4′-methyl-2,2′-bipyridine (1.5 g, 5.6 mmol) and conc. HCl (35–37%, 10 mL) was stirred at 100 °C for 15 h. The resulting mixture was cooled down to room temperature and adjusted the pH value to 4.0–4.5 with 6 N NaOH and extracted with chloroform. The organic phase was washed with brine, dried over $MgSO_4$ and concentrated in *vacuo* to yield 6-(4′-methyl-2,2′-bipyridin-4-yl)hexanoic acid (1.4 g, 86%) as a magenta powder. 6-(4′-Methyl-2,2′-bipyridin-4-yl)hexanoic acid (280 mg, 2.0 mmol) was dissolved in 25 mL DMSO in a flask. DMT-MM (550 mg, 2.0 mmol) was added to the solution and stirred at room temperature for 1 h, followed by addition of Amino-POSS (1.2 g, 1.0 mmol) and triethylamine (280 μL, 3.1 mmol). The mixture was stirred at 25 °C for 51 h and dropped into 250 ml acetonitrile containing 0.2 wt% HCl and the products were precipitated. The precipitation was collected by filtration and washed with acetonitrile and dried *in vacuo*. The resultant solid was dissolved in methanol and the precipitation step was repeated. The pale magenta powder of bpyL-POSS was thus obtained (1.2 g, 0.89 mmol, 89%). The average substitution rate with the bpy ligands was 1.0 of eight aminopropyl moieties of Amino-POSS, which is calculated from the integration ratios of the ^{1}H NMR peaks.

Preparation of ruthenium(II) complex substituted with POSS (Ru-POSS). A typical procedure is shown here. A mixture containing 0.40 g of bpyL-POSS (bipyridine unit; 0.28 mmol) and Ru(II)$(bpy)_2Cl_2$ (130 mg, 0.28 mmol) were heated at 85 °C in 8 mL aqueous ethanol solution (ethanol/water = 1/3 v/v) for 37 h. The resulting solution was poured into acetonitrile containing 0.1 wt% HCl, and the sticky sediment was separated from supernatant by decantation. The obtained precipitate was washed with acetonitrile three times by ultrasonic. The crude product was dissolved in methanol, and the solvent was removed by a rotary evaporator and *in vacuo*. The title compound was obtained as a dark red powder (0.35 g, 0.21 mmol, 71%). ^{1}H NMR (Chart S1, 400 MHz, DMSO-d_6, 25 °C): δ 8.85 (br, 4.1H), 8.24−8.14 (m, 25H), 7.75−7.69 (m, 2.6H), 7.55−7.49 (m, 4.1H), 7.39−7.35 (m, 1.5H), 2.80 (br, 17H), 2.12 (br,1.4H), 1.72 (br, 16H), 1.50 (br, 3.3H), 1.28 (br, 1.4H), 0.70 (br, 16H) ppm. ^{13}C NMR (Chart S2, 100 MHz, CD_3OD , 25 °C): δ 176.3, 158.6, 158.6, 158.2, 158.0, 156.3, 155.3, 155.1, 154.7, 154.5, 152.6, 152.1, 151.9, 151.6, 139.0, 129.7, 129.0, 128.9, 126.6, 125.8, 125.6, 43.1, 36.8, 36.0, 30.9, 30.0, 26.8, 24.0, 22.3, 21.3, 10.3, 9.6 ppm. The peaks between δ 156 ppm and δ 154 ppm are for reference purpose only due to noisy baseline originated from an enormous accumulation number, which is needed for detecting signals weakened by substitution with Ruthenium. ^{29}Si NMR (Chart S3, DMSO-d_6, 25 °C): δ −66.6 ppm. The average substitution rate with ruthenium unit (R^2 in Scheme 1) is 0.7 of eight aminopropyl moieties of Amino-POSS, estimated from the integration ratios of the ^{1}H NMR peaks basically at the bipyridine ligand as shown in Chart S1. CryoSpray MS (N_2: −20 °C) $[M]^{2+}$ calcd. 780.2312, found 780.2319.

Preparation of a model compound (Ru-Model). 6-(4′-Methyl-2,2′-bipyridin-4-yl)hexanoic acid (200 mg, 0.70 mmol) was dissolved in 10 mL DMSO in a Schlenk flask. DMT-MM (390 mg, 1.4 mmol) was added to the solution and stirred at room temperature for 90 min, followed by addition of propylamine (200 μL, 2.4 mmol) and triethylamine (200 μL, 1.4 mmol). The mixture was stirred at 25 °C for 39 h and then mixed with 200 mL hexane/ethyl acetate/water (4/1/4 v/v/v) and the organic phase was collected and washed twice with water. The organic phase was dehydrated with Na_2SO_4, evaporated to dryness and dissolved into methanol. The obtained solution was purified by silica gel column chromatography (hexane/ethyl acetate = 1/3 v/v containing 1 v/v % trimethylamine to give 73 mg (32%) white powder, 6-(4′-methyl-[2,2′-bipyridin]-4-yl)-N-propylhexanamide. 40 mg (0.12 mmol) of this bipyridine derivative and Ru(II)$(bpy)_2Cl_2$ (59 mg, 0.12 mmol) were added into 8 mL EtOH/water (1/3 v/v) solution and then refluxed for 24 h. The

resultant solution was filtrated and evaporated for dryness and then mixed with hexane/ethyl acetate/water (4/1/4 v/v/v). The aqueous phase was evaporated for dryness to give a dark red solid (87 mg, 88%). ^{1}H NMR (Chart S4, DMSO-d_6, 25 °C): δ 8.88 (s, 2H), 8.86 (s, 3H), 8.78 (s, 1H), 8.18–8.15 (m, 4H), 7.87 (t, J = 5.5 Hz, 1H), 7.76–7.69 (m, 4H), 7.58–7.51 (m, 6H), 7.38 (t, J = 6.8 Hz, 2H), 2.94 (q, J = 6.6 Hz, 2H), 2.76 (t, J = 7.6 Hz, 2H), 2.52 (s, 3H), 2.07 (t, J = 7.2 Hz, 2H), 1.68 (quint, J = 7.6 Hz, 2H), 1.53 (quint, J = 7.5 Hz, 2H), 1.38–1.25 (m, 4H), 0.75 (t, J = 7.4 Hz, 3H) ppm. ^{13}C NMR (Chart S5, 100 MHz, DMSO-d_6, 25 °C): δ 171.8, 156.6, 156.6, 156.1, 156.0, 153.9, 151.2, 151.1, 151.1, 150.9, 150.4, 150.2, 149.7, 137.7, 128.5, 127.8, 125.3, 124.4, 124.4, 35.1, 34.2, 29.1, 28.1, 24.9, 22.4, 20.6, 11.3 ppm. ESIMS $[M]^{2+}$ calcd. 369.6281, found 369.6286.

References

(1) Jeon, J.-H.; Tanaka, K.; Chujo, Y. *Org. Biomol. Chem.* **2014**, *12*, 6500–6506.

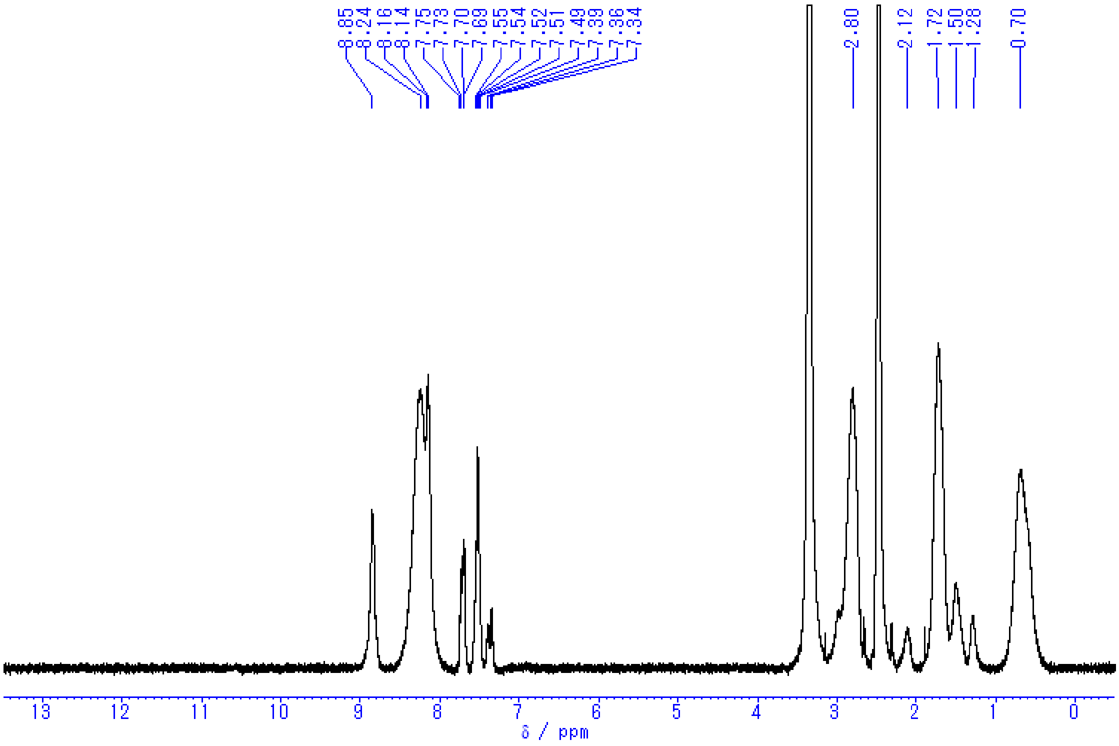

Chart S1. ^{1}H NMR spectrum of Ru-POSS in DMSO-d_6 at 25 °C (400 MHz).

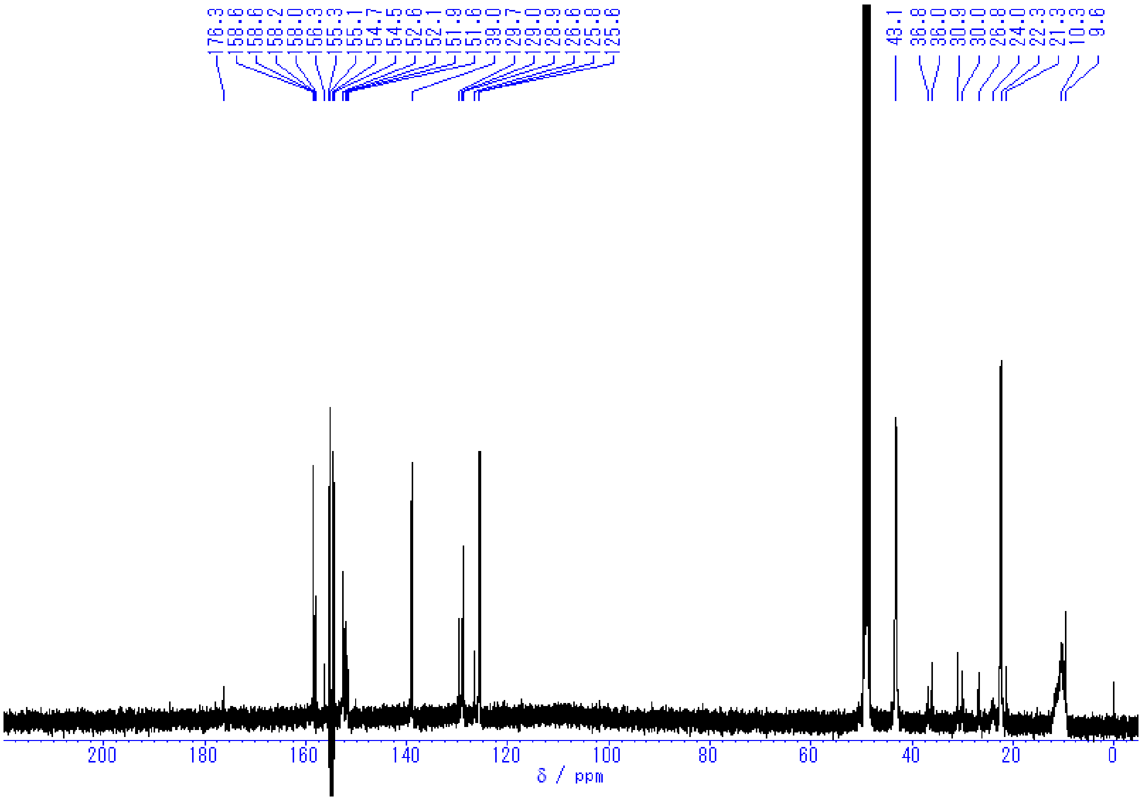

Chart S2. ^{13}C NMR spectrum of Ru-POSS in CD_3OD at 25 °C (100 MHz).

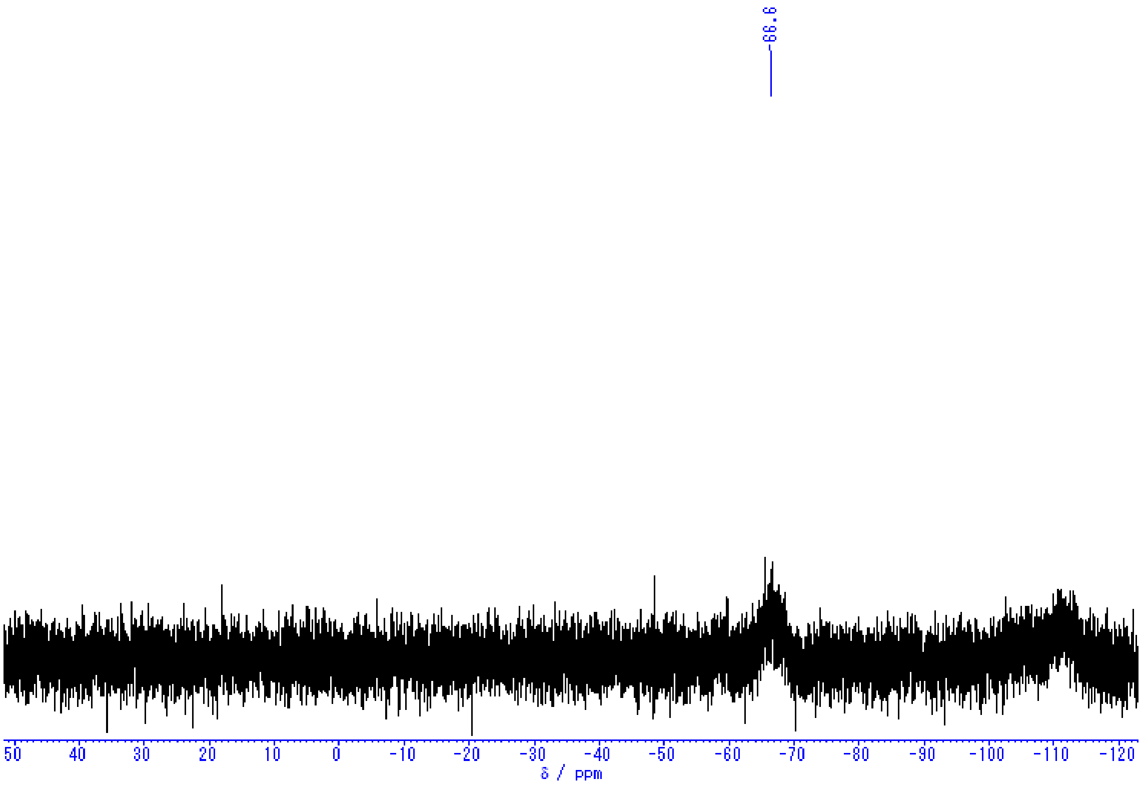

Chart S3. ^{29}Si NMR spectrum of Ru-POSS in DMSO-d_6 at 25 °C (80 MHz).

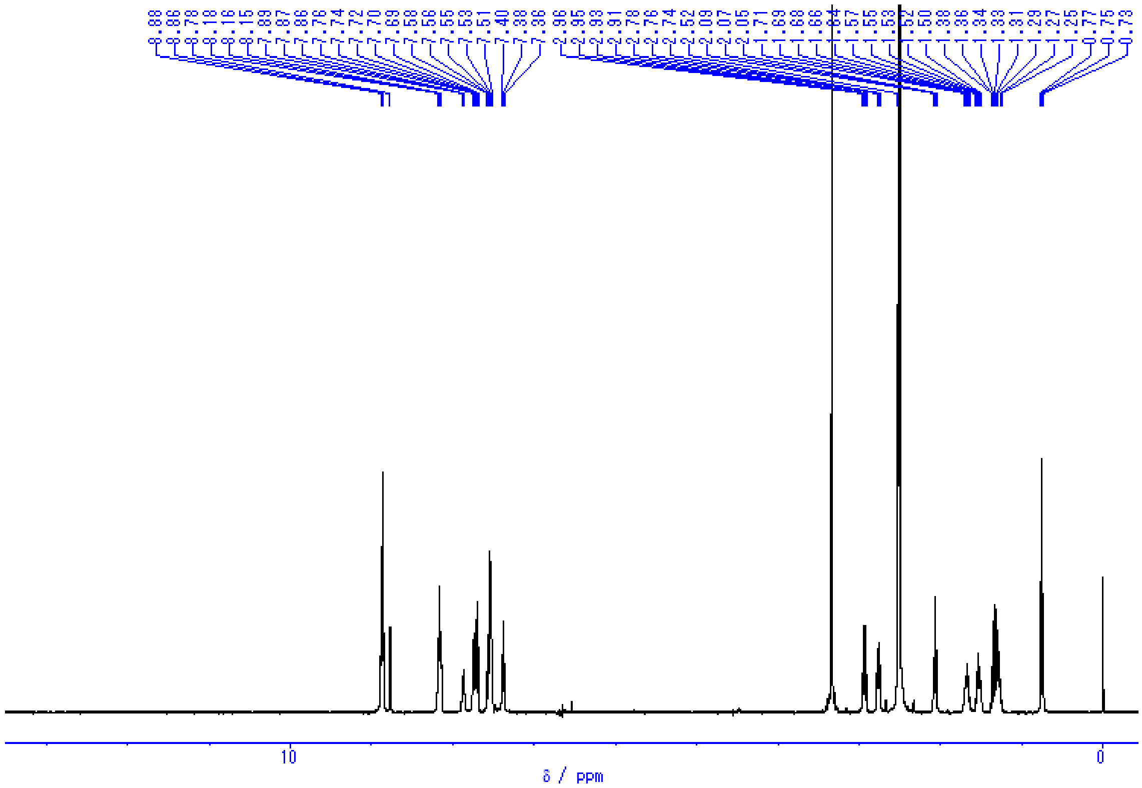

Chart S4. ^{1}H NMR spectrum of Ru-Model in DMSO-d_6 at 25 °C (400 MHz).

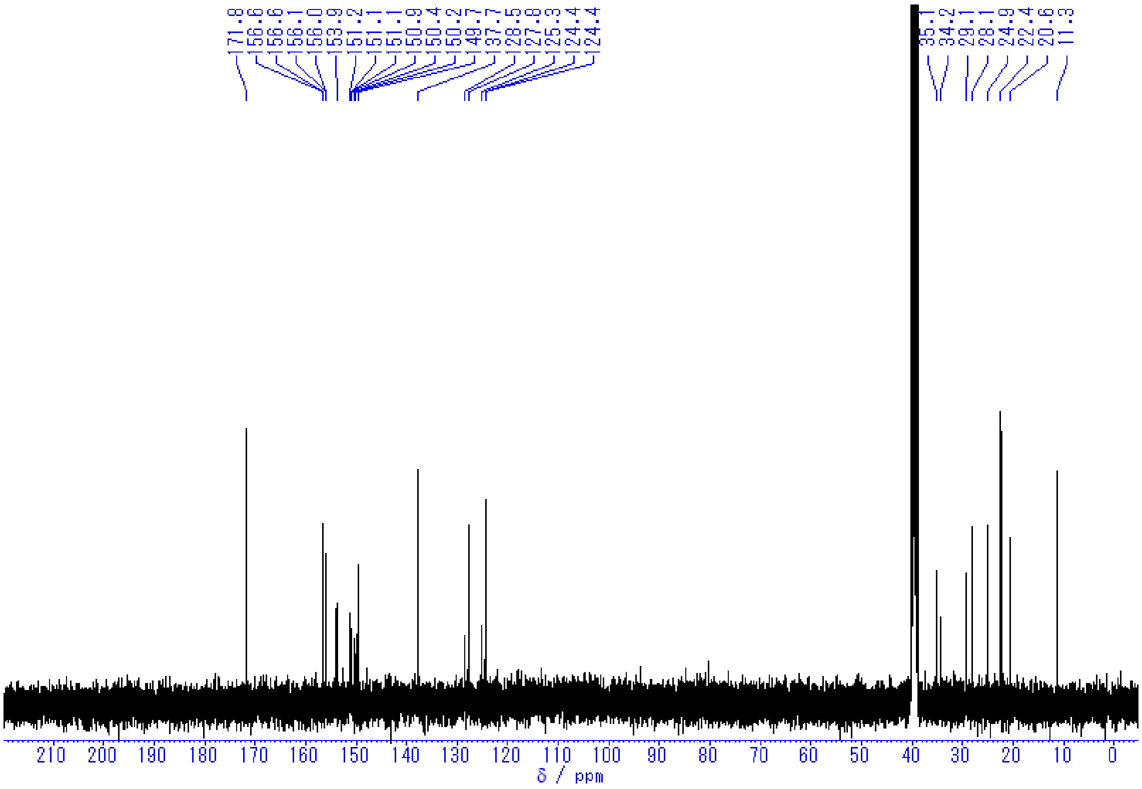

Chart S5. ^{13}C NMR spectrum of Ru-Model in DMSO-d_6 at 25 °C (100 MHz).

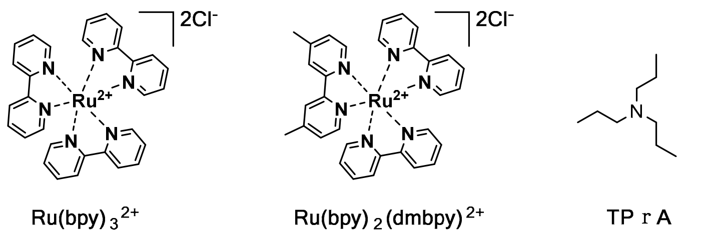

Figure S1. Structures of $Ru(bpy)_3^{2+}$, $Ru(bpy)_2(dmbpy)^{2+}$ and TPrA.

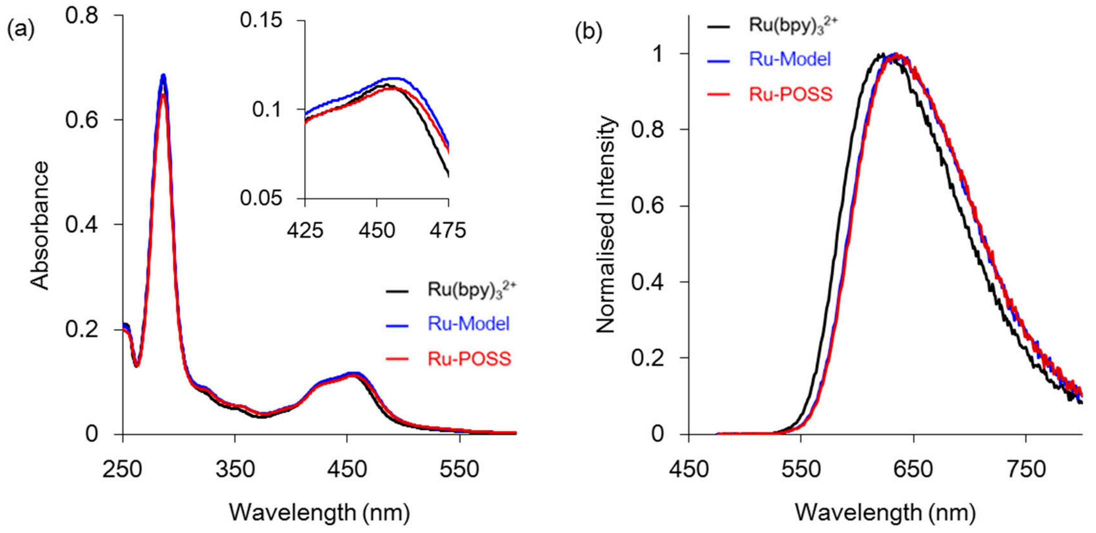

Figure S2. (a) UV−vis absorption and (b) PL spectra of 1.0×10^{-5} M solutions containing $Ru(bpy)_3^{2+}$, Ru-Model and Ru-POSS in H_2O. The excitation light at λ_{abs_MLCT} was used for PL measurements. Inset figure in (a) is the inset around the MLCT band. The concentration of Ru-POSS was based on the Ru(II) complex unit.

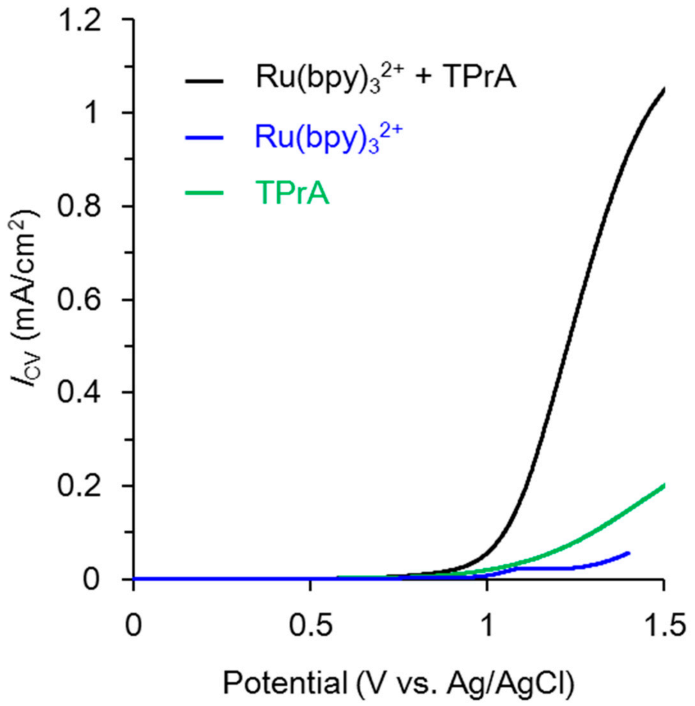

Figure S3. Cyclic voltammograms of 0.10 mM Ru(bpy)$_3^{2+}$ and/or 0.1M TPrA in 0.20 M PBS buffer (pH 8.8) at ITO electrode at a scan rate of 100 mV/s (n = 3).

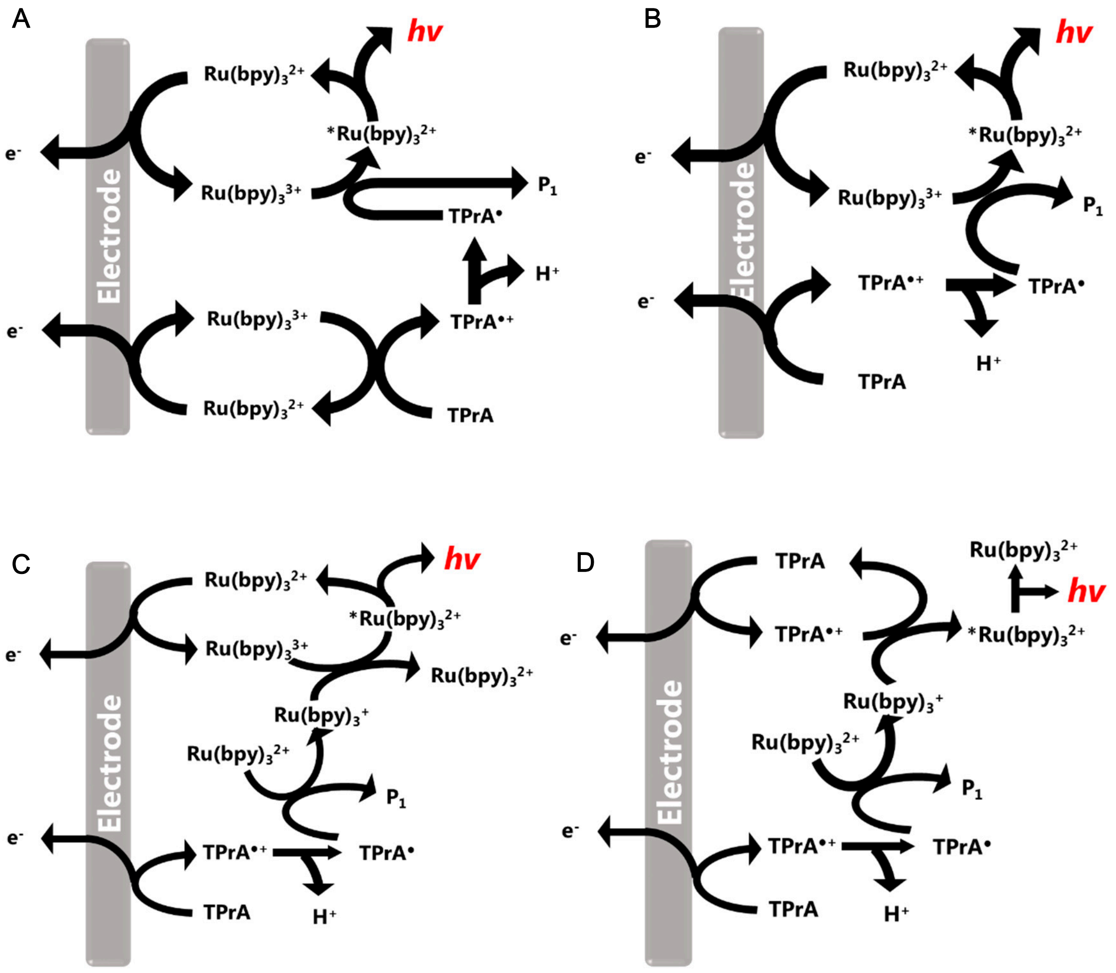

Scheme S1. The summary of the ECL mechanisms in the $Ru(bpy)_3^{2+}$/TPrA system ($TPrA^{\bullet+} = Pr_3N^{\bullet+}$, $TPrA^{\bullet} = Pr_2NC^{\bullet}HCH_2CH_3$, $P_1 = Pr_2N^{+}CHCH_2CH_3$).

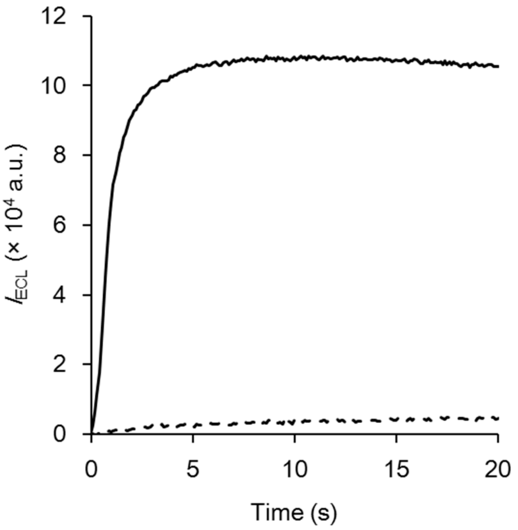

Figure S4. Time courses of ECL in BE of 0.50 mM $Ru(bpy)_3^{2+}$ and 100 mM TPrA in 0.20 M PBS buffer (pH 8.8) with deoxygenation (solid line) or aeration (dashed line) with an ITO electrode at 1.2 V vs. Ag/AgCl.

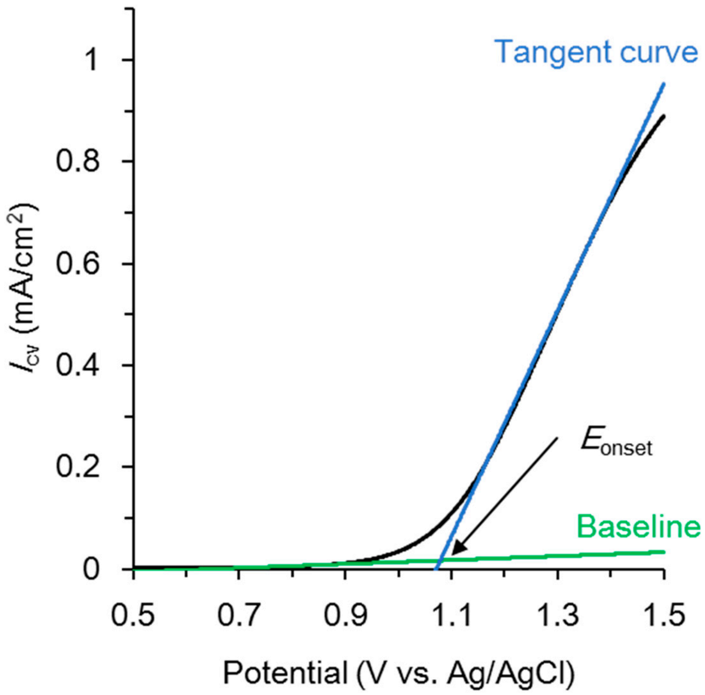

Figure S5. The determination of onset potential (E_{onset}). The onset potentials were determined from the intersection of the tangents between the baseline and the signal current.

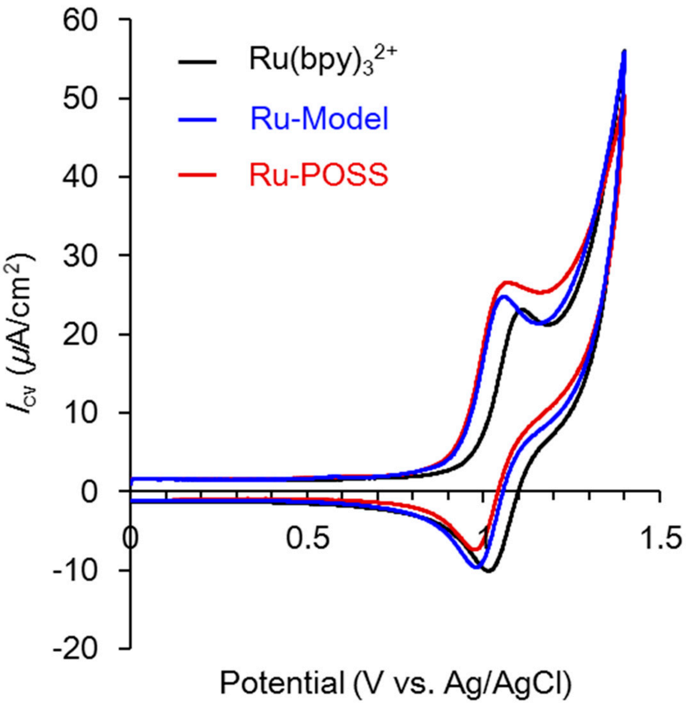

Figure S6. Cyclic voltammograms of 0.10 mM Ru(II)-containing materials in 0.20 M PBS buffer (pH 8.8) with an ITO electrode at a scan rate of 100 mV/s (n = 3). The concentration of Ru-POSS was based on the Ru(II) complex unit.

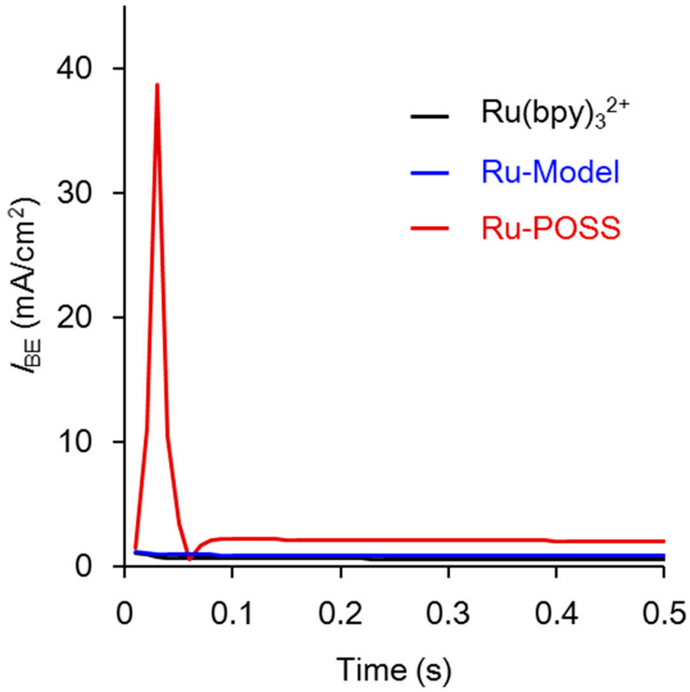

Figure S7. Initial time courses of current in BE of 0.10 mM Ru(II) complexes and 100 mM TPrA in 0.20 M PBS buffer (pH 8.8) at ITO electrode at 1.2 V vs. Ag/AgCl (n = 3). The concentration of Ru-POSS was based on the Ru(II) complex unit.

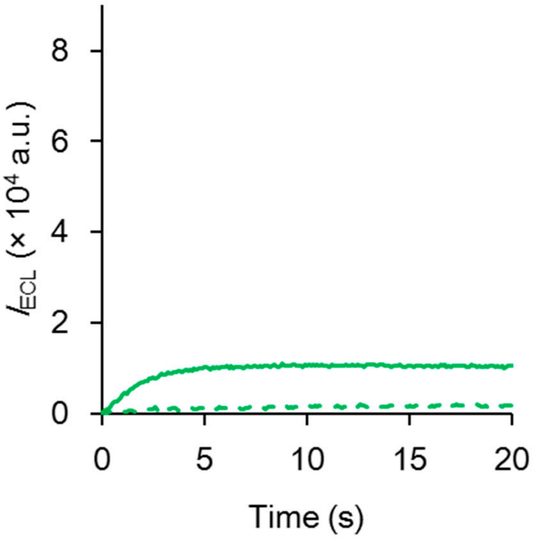

Figure S8. Time courses of ECL with 0.1 mM $Ru(bpy)_3^{2+}$, 0.1 mM Amino-POSS and 100 mM TPrA in 0.20 M PBS buffer (pH 8.8) with Ar (solid line) and O_2 bubbling (dashed line) with an ITO electrode at 1.2 V vs. Ag/AgCl.

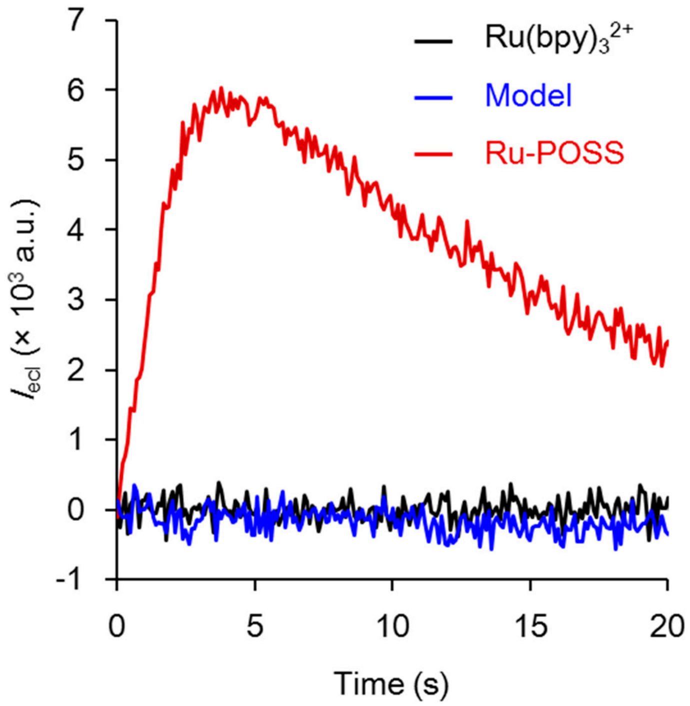

Figure S9. Time courses of ECL in BE of 100 mM TPrA in 0.20 M PBS buffer (pH 8.8) with the modified ITO electrodes at 1.2 V vs. Ag/AgCl.

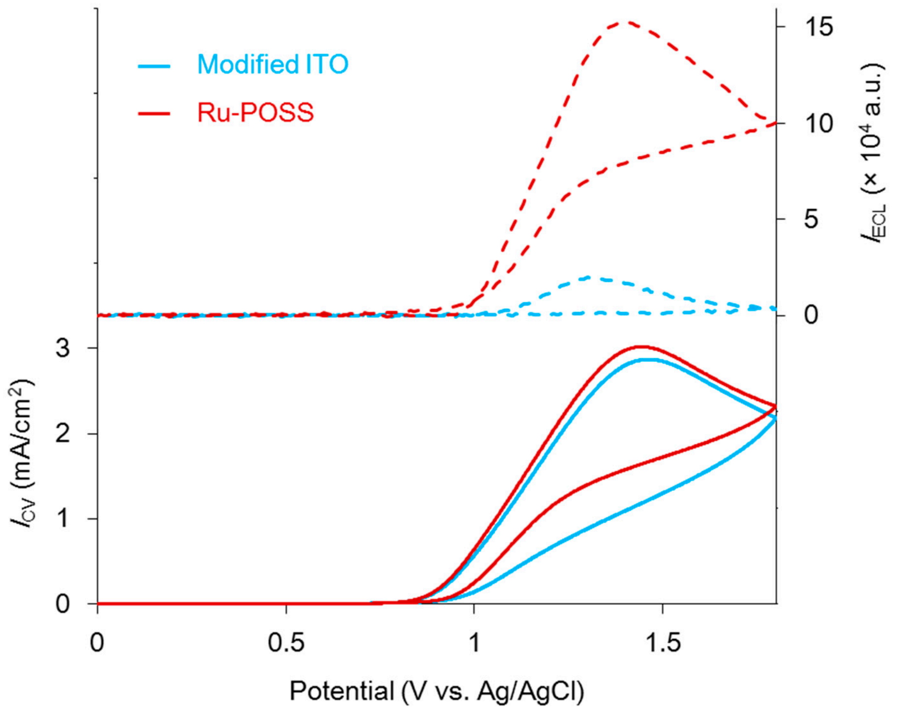

Figure S10. Cyclic voltammograms (solid lines) and corresponding ECL curves (dashed lines) at a scan rate of 100 mV/s with 0.1 mM Ru-POSS and 100 mM TPrA in 0.20 M PBS buffer (pH 8.8) with an ITO electrode (Red line) and 100 mM TPrA in 0.20 M PBS buffer (pH 8.8) with the modified ITO electrode (Blue line). The concentration of Ru-POSS was based on the Ru(II) complex unit.

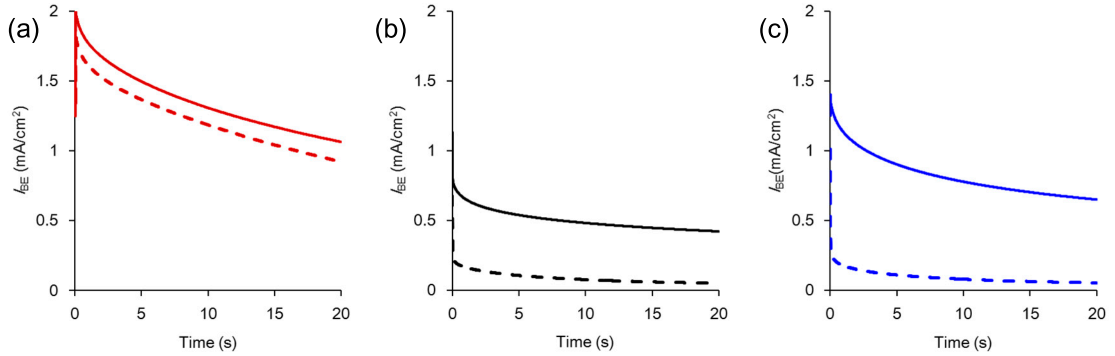

Figure S11. Time courses of currents with (a) Ru-POSS (b) $Ru(bpy)_3^{2+}$ (c) Ru-Model in BE of 100 mM TPrA in 0.20 M PBS buffer (pH 8.8) with (solid line) or without 0.10 mM Ru(II) complexes (dashed line) at ITO electrode at 1.2 V vs. Ag/AgCl (n = 3). The concentration of Ru-POSS was based on the Ru(II) complex unit.

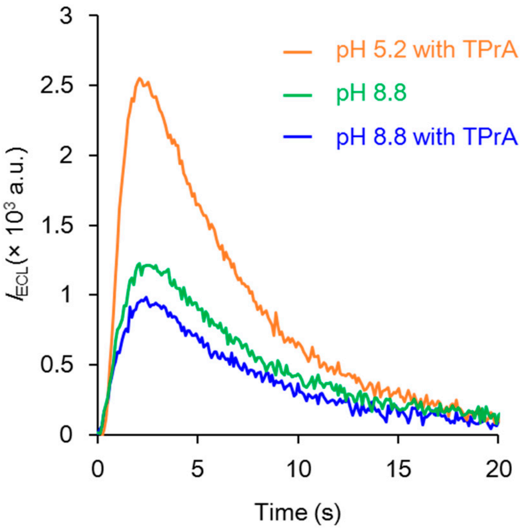

Figure S12. Time courses of ECL in BE of 100 mM TPrA in 0.20 M PBS buffer (pH 8.8) at 1.2 V vs. Ag/AgCl with the modified ITO electrodes immersed into the solution containing 0.1 mM Ru-POSS (based on the Ru(II) complex unit) with 100 mM TPrA in 0.20 M PBS buffer with variable pH values (pH 5.2: orange line and pH 8.8: blue line).

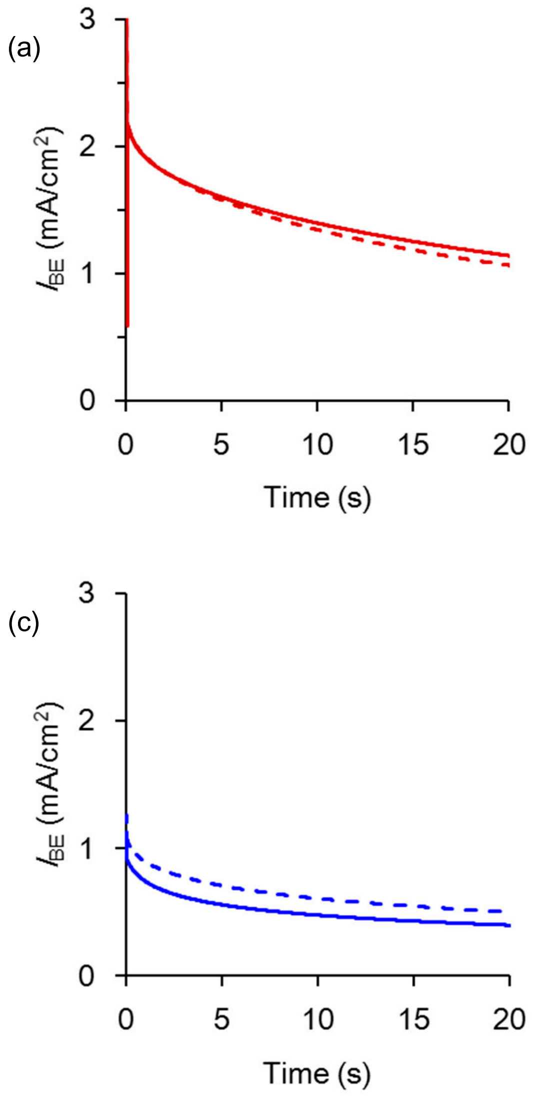

Figure S13. Time courses of electric currents with (a) Ru-POSS, (b) $Ru(bpy)_3^{2+}$, (c) Ru-Model and (d) $Ru(bpy)_3^{2+}$ + Amino-POSS in BE of 0.10 mM Ru(II) complexes and 100 mM TPrA in 0.20 M PBS buffer (pH 8.8) with Ar (solid line) and O_2 bubbling (dashed line) with and ITO electrode at 1.2 V vs. Ag/AgCl. The concentration of Ru-POSS was based on a Ru(II) complex unit. The concentration of Amino-POSS in (d) was adjusted to the same as the POSS unit in (a).

Table S1. Residual rates of maximum ECL intensities and onset increase rates in BE in the aerated solution compared to the hypoxic one

	$I_{ECL}^{Max}{}_{\_O2}/I_{ECL}^{Ma}x_{\_Ar}$ (%)	$m_{ECL}^{Max}{}_{\_O2}/m_{ECL}^{Ma}x_{\_Ar}$ (%)
$Ru(bpy)_3^2$ + Amino-POSS	25	10

* *m*: Slope of onset intensity curve

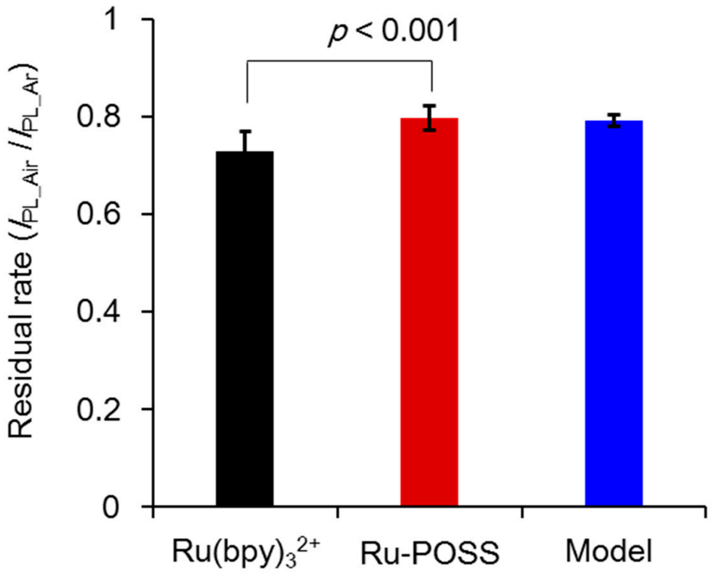

Figure S14. Residual rates of emission intensities of the photo-excited Ru(II) complexes in aerated solutions consist of 0.01 mM Ru(II) complexes and 0.1 M TPrA in 0.2 M PBS solution (pH 8.8). Excitation wavelengths were λ_{abs_MLCT} Residual rates were calculated from the equation, I_{PL_Air} / I_{PL_Ar}, where I_{PL_Air} is the intensity at λ_{em}^{max} in aerated solutions and I_{PL_Ar} is in the hypoxic solutions.

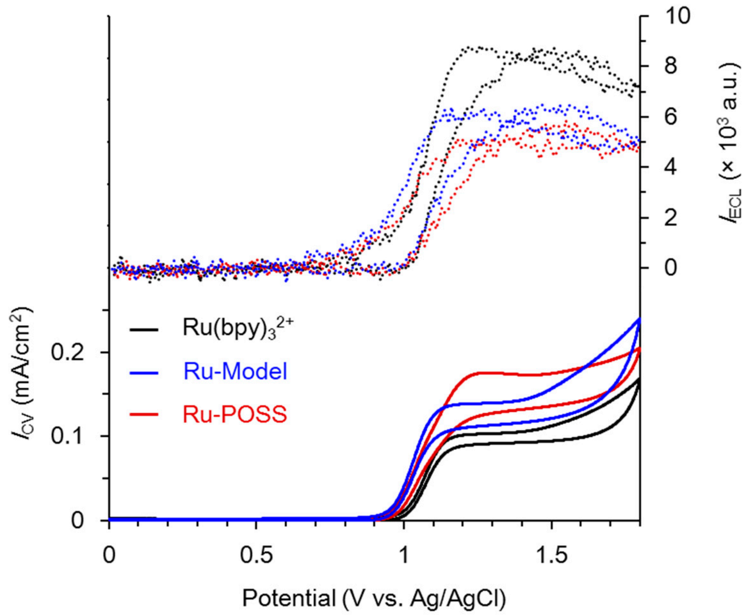

Figure S15. Cyclic voltammograms (solid lines) and corresponding ECL curves (dotted lines) of 0.1 mM Ru(II) complexes and 100 mM TPrA in 0.20 M PBS buffer (pH 5.2) with an ITO electrode at a scan rate of 100 mV/s (n = 3). The concentration of Ru-POSS was based on a Ru(II) complex unit.

Table S2. E_{onset} in various conditions[a]

		Ru-POSS	Ru-Model	$Ru(bpy)_3^{2+}$
Ru(II) complex[a] with TPrA[b]	pH 8.8	0.92	1.00	1.08
	pH 5.2	0.97	0.97	1.00
Ru(II) complex[c]	pH 8.8	0.94	0.94	0.99
	pH 5.2	0.95	0.95	0.99

[a] Measured in 0.20 M PBS buffer at ITO electrode at a scan rate of 100 mV/s (n = 3).

[b] 0.1 M.

[c] 0.1 mM.
